# Challenges in Management of Primary Hypoparathyroidism Associated with Autoimmune Polyglandular Syndrome Type 1

**DOI:** 10.1155/2011/281758

**Published:** 2011-09-07

**Authors:** I. R. Wallace, V. McConnell, P. M. Bell, J. R. Lindsay

**Affiliations:** ^1^Department of Endocrinology and Diabetes, Altnagelvin Area Hospital, Londonderry BT47 6SB, UK; ^2^Northern Ireland Regional Genetics Service, Belfast City Hospital, Belfast BT9 7AB, UK; ^3^Regional Centre for Endocrinology and Diabetes, Royal Victoria Hospital, Grosvenor Road, Belfast BT12 6BA, UK

## Abstract

We report a case of autoimmune polyglandular syndrome type 1 (APS1) complicated by severe vascular insufficiency due to diffuse vascular calcification. APS1 is characterised clinically by multiple autoimmune conditions and development of at least two components of the triad of mucocutaneous candidiasis, hypoparathyroidism, and autoimmune adrenal insufficiency. We highlight the problems in current serum calcium monitoring methods and suggest that fluctuations in serum calcium concentrations due to difficulties treating hypoparathyroidism may have contributed to the vascular calcification seen in this case.

## 1. Introduction

Autoimmune polyglandular syndrome type 1 (APS1) is a rare condition with autosomal recessive inheritance. It is characterised clinically by multiple autoimmune conditions and development of at least two components of the triad of mucocutaneous candidiasis, hypoparathyroidism, and autoimmune adrenal insufficiency. The following case highlights some of the challenges and complications encountered in managing hypoparathyroidism in this setting.

## 2. Case Presentation

**Figure 1:**
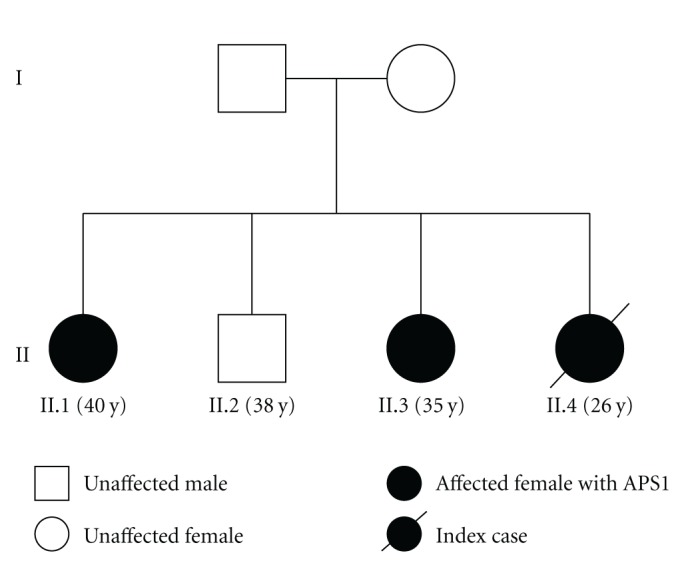
Pedigree.

Our index Case (II.4) was the youngest sibling from a family of four living in the North West region of Northern Ireland born to nonconsanguineous parents (Figure 1). Three of the sibship (all female) who had presented with a variable range of clinical manifestations of APS1 (Table 1) were subsequently shown to be homozygous for a 13 base pair (bp) deletion in exon 8 (c.964del13) in the autoimmune regulator gene (AIRE-1). The other sibling (II.2) was clinically unaffected and has not undergone carrier genetic testing for APS1. Cases II.1 and II.3 are undergoing regular medical followup, with their clinical features summarised in Table 1.

### 2.1. Index Case

Our index Case (II.4) was diagnosed with APS1 in childhood, presenting with mucocutaneous candidiasis at age 5. Hypoparathyroidism was diagnosed at age 8 following an admission due to a seizure associated with hypocalcaemia. At age 10 autoimmune adrenal insufficiency was confirmed and treatment commenced with hydrocortisone and fludrocortisone. Serum potassium concentration remained within the reference range. Type 1A Diabetes Mellitus was diagnosed at age 18. Glycaemic control was suboptimal with a number of admissions due to diabetic ketoacidosis, and HbA1c never below 8%. She also developed proliferative retinopathy and underwent vitrectomy following retinal hemorrhage. She developed multiple features of APS1, which are summarised in Table 1.

**Figure 2:**
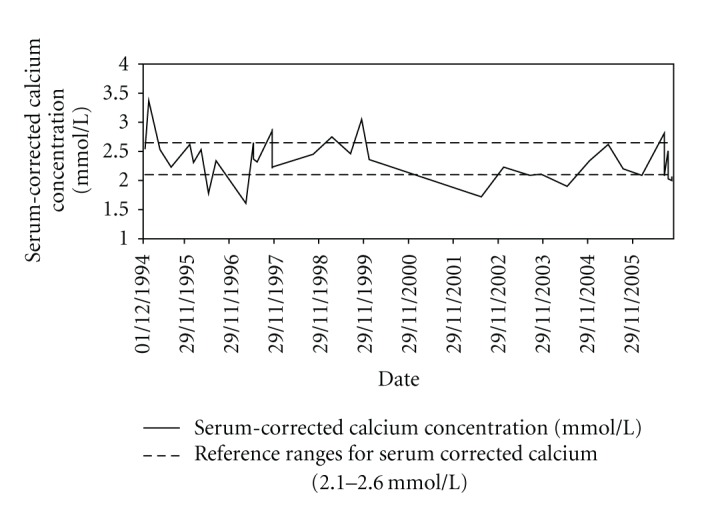
Chart showing variations in serum-corrected calcium concentrations (mmol/L) over time for Case II.4.

Hypoparathyroidism was treated with oral Alfacalcidol titrated according to serum corrected calcium concentrations.  Figure 2 illustrates variations in serum corrected calcium concentrations over time. Urinary calcium excretion was measured intermittently with values ranging from 2.19–4.80 mmol/24 hrs. In the last year of her life, estimated glomerular filtration rate was between 30 and 45 mL/min and serum phosphate between 1.35 and 1.55 mmol/L. She was not treated with a phosphate binder. 

**Figure 3:**
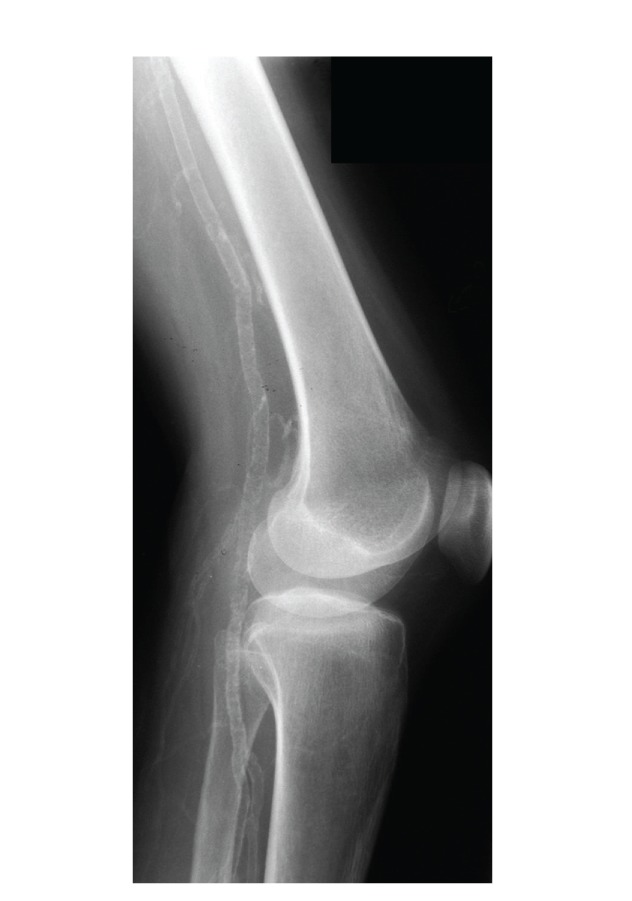
Plain radiograph of right leg showing diffuse vascular calcification of the femoral and popliteal arteries.

Her subsequent progress was complicated by recurrent admissions with generalised tonic-clonic seizures and hypocalcaemia. In 2003, she presented with acute ischaemia of the distal tip of her left 5th finger, and in 2004, she was admitted for observation following a collapse episode associated with QTc prolongation on ECG, which was successfully treated with intravenous calcium gluconate with normalisation of the QTc interval. Her progress was complicated by renal calculi, diffuse nephrocalcinosis, and chronic renal failure. In 2006, she complained of intermittent claudication at a distance of 20 yards. Dorsalis pedis and posterior tibial pulses were diminished bilaterally with ankle-brachial pressure indexes >1.0 consistent with vessel calcification. Diffuse large vessel calcification was evident on a plain radiograph of the right leg (Figure 3). She subsequently developed ulceration around the left great toe, progressing to bilateral lower limb critical ischaemia and rest pain. Magnetic resonance (MR) angiogram of the lower limb vessels showed diffuse vascular calcification, but no focal stenotic lesions were identified. In July 2006, due to ongoing ischaemia her left great toe became necrotic with proximal spread. A conservative treatment plan was agreed, as she was too unwell to proceed to surgery. Pain relief and palliation was achieved using opiates, given subcutaneously by syringe driver and intrathecally. In October 2006, she died, aged 26 years old, as a result of fulminant sepsis secondary to infected gangrene of the left foot.

## 3. Discussion

APS1 is a rare disorder in most populations, with an estimated incidence of 1 in 25,000 in Finland. It is more common in females and is inherited in an autosomal recessive manner. Mutations in the AIRE gene, which encodes a transcription factor cause the syndrome. The AIRE gene is located on chromosome 21 [1]. Over 50 mutations have been reported worldwide but there are common mutations specific to different APS1 patient groups. The 13 base-pair deletion in exon 8 (964del13) found in our case was present in 70% of alleles in a sample of British APS1 patients [2]. The majority of the AIRE-1 mutations are predicted to cause a truncation of the protein, which is consistent with a loss of AIRE-1 function leading to APS1 [3]. The exact role of AIRE-1 in regulation of immune responses is unknown. It has a high degree of interfamilial and intrafamilial phenotypic variability. Genotype-phenotype correlation is not possible due to the extensive evidence of intrafamilial phenotypic variability. 

Vascular calcification results when the normal balance between factors promoting and inhibiting vascular calcification is disturbed. Four distinct but overlapping forms are described. These are atherosclerotic calcification, medial artery calcification, cardiac valve calcification, and calciphylaxis [4]. It is suggested that cells may be induced to differentiate into osteoblast-like cells which will secrete and deposit extracellular osteoid matrix. Candidate cells may include stem cells, vascular smooth muscle cells, and pericytes. Proposed triggers for this change include bone morphogenetic protein, oxidative stress, hyperphosphatemia, vitamin D, and parathyroid hormone. Proposed inhibitors include pyrophosphate, osteopontin, osteotegrin, and fetuin. Vascular calcification, particularly medial artery, and atherosclerotic calcification is common in the presence of diabetes mellitus and chronic renal failure [5]. Arterial calcification in the presence of APS1 has previously been reported [6].

The present case highlights some of the challenges in the treatment of hypoparathyroidism in APS I. The goal of management of hypoparathyroidism is to alleviate symptoms of hypocalcaemia and avoid complications of hypercalcaemia by maintaining serum calcium concentrations in the low normal range [4]. 

APS I presents specific challenges in achieving eucalcaemia due to other manifestations of APS I including intestinal malabsorption, coeliac disease, pancreatic exocrine failure, or intestinal lymphangiectasia. Variable PTH reserve and secretion may also lead to calcium excursions and ectopic calcification. Careful monitoring of serum and urinary calcium levels is required in all patients [7].

In our case, serum calcium values were usually in the target low-normal range during followup (Figure 2). Nevertheless, she still developed diffuse vascular calcification, illustrating the limitations of current therapy, which is nonphysiological. Intermittent monitoring of serum calcium levels may have been insufficiently sensitive to detect serum calcium excursions as this method provides a point estimate rather than an integrated measure of current calcium balance. We recognise that the concomitant presence of Type 1A diabetes mellitus of 8 years duration and chronic renal failure probably contributed to her early peripheral vascular disease; however, the severity of her presentation at a young age and rapidly progressive clinical course suggest that fluxes in calcium homeostasis probably played an important role in her progressive deterioration.

In conclusion, patients with APS 1 are recognized to be potentially at risk of premature death due to adrenal crisis, hypocalcaemia, or severe sepsis. Diffuse ectopic calcification and vascular insufficiency is unexpected but has previously been reported [6]. The fatal clinical course in our index Case (II.4) illustrates the complexity of polyglandular disease, some of the challenges in managing hypoparathyroidism in patients with Type 1 diabetes mellitus in APS 1, and the importance of monitoring these patients with appropriate and prompt intervention. The intrafamilial and interfamilial phenotypic variability further complicates and will have implications for the affected siblings of our index case and in particular II.3. Our family highlights the paucity of genotype-phenotype correlation in APS 1 with the degree of intrafamilial variability observed at this stage.

## Figures and Tables

**Table 1:** Phenotypic manifestations of APS1 in affected family members.

Phenotype	II.1	II.3	II.4
Mucocutaneous candidiasis	+	+	+
Esophageal candidiasis		+	+
Primary hypoparathyroidism	+	+	+
Primary adrenal insufficiency	+	+	+
Pernicious anaemia		+	+
Vitiligo		+	+
Type I diabetes mellitus		+	+
Premature ovarian failure	+	+	+
Pancreatic exocrine insufficiency		+	+
